# Single-port splenectomy: Current update and controversies

**DOI:** 10.4103/0972-9941.72383

**Published:** 2011

**Authors:** Eduardo M Targarona, Maria B Lima, Carmen Balague, Manuel Trias

**Affiliations:** Service of Surgery, Hospital de Sant Pau, University Autonomous of Barcelona, Barcelona, Spain

**Keywords:** Endoscopic surgery, literature review, single-port access, splenectomy, surgical technique

## Abstract

Multiport laparoscopic splenectomy (LS) is considered the “gold standard” for the management of surgical diseases in normal or slightly enlarged spleens. The concept of minimal-invasive surgical techniques has progressed since the early 1990s from standard multiport laparoscopy to natural orifice transluminal endoscopic surgery (NOTES) and, more recently, to single-port access (SPA). In this paper, we describe our technique for SPA splenectomy and provide a critical review of the current literature on SPA for splenic diseases.Preliminary results published to date indicate that the spleen can be safely removed using single-incision surgery and all the authors have unanimously endorsed the feasibility of this approach. However, available evidence is still scarce. It is based only on case reports and one small series, with a total of 17 patents and, therefore, firm conclusions cannot yet be drawn and more experience and comparative trials are needed to determine the exact role of this interesting new approach.

## INTRODUCTION

Multiport laparoscopic splenectomy (LS) is considered the “gold standard” for the management of surgical diseases in normal or slightly enlarged spleens. Its effectiveness and low-complication rate, alongside patient comfort, decreased hospital stay and enhanced recovery make it the procedure of choice among doctors and patients.[1]

The concept of minimal-invasive surgical techniques has progressed since the early 1990s, from standard multiport laparoscopy to natural orifice transluminal endoscopic surgery (NOTES) and, more recently, to single-port access (SPA). Experience with SPA has been reported sporadically since minimal-invasive procedures (appendectomy, cholecystectomy) first appeared, but the number of papers on the subject has increased consistently since 2007, perhaps because surgeons view this technique as a bridge to the even lesser-invasive NOTES.[2,3] In this paper, we describe our technique for SPA splenectomy and provide a critical review of the current literature on SPA for splenic diseases.

## SURGICAL TECHNIQUE

**Figure 1:**
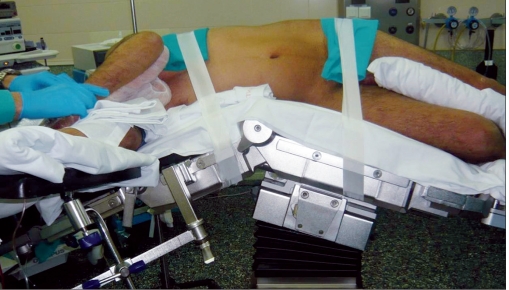
Patient position: full lateral decubitus

Surgical technique has been published elsewere.[4,5] The patient is placed in the standard right decubitus position for LS, with the table flexed at the flank [Figure 1]. The transumbilical approach can be chosen for thin patients and in cases of splenic cyst. In the case of a slightly enlarged spleen, a left 2 cm subcostal incision is placed at a point between the subcostal margin and the umbilicus in the midclavicular line. We use one of two approaches. (1) SPA splenectomy using multiple trocars: a 15-mm skin incision is made and, after establishing pneumoperitoneum, a 12-mm bladeless trocar (Excel Endopath, Ethicon Endo-Surgery, Cincinnati, OH, USA) is bluntly introduced into the abdomen under optic control with a flexible tip 10 mm HD scope (Olympus, Center Valley, PA, USA). After exploring the abdominal cavity, a 5-mm trocar with a flexible corrugate shaft (Karl Storz, Culver City, CA, USA) is inserted into the left of the 12-mm trocar and another 5-mm trocar with a small head is placed to the right. (2) SAP splenectomy using a multiport device [Figure 2a]: after the insertion of the veress needle, a 20-mm incision is made and a multiple-port device (Triport, Quadriport, [Olympus], Uno [Ethicon]) is inserted.

**Figure 2a:**
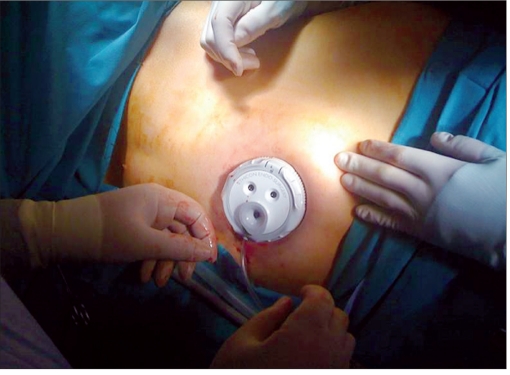
(a) Uno device (Ethicon) located at the umbilicus.

The technique used for splenic dissection is similar to that used in standard LS. After an explorative laparoscopy, the possible existence of accessory spleens ruled out and a 5-mm curved grasper is used for transanal endoscopic microsurgery (TEM) (Richard Wolff, Vernon Hills, IL, USA) is placed through the left port. The slightly curved end of this instrument fits into the flexible trocar or through a port of the mutichannel device, and it is suffciently curved to work intrabdominally without causing instruments to clash. A 5-mm harmonic scalpel (Harmonic Ace, Ethicon Endo-Surgery, Cincinnati, OH, USA) is then introduced through the right port. Using this approach, it is possible to mobilize the splenic colon flexure and to reach the lower pole of the spleen. The next step is to gain access to the retrogastric pouch and to section the short vessels at the upper pole of the spleen. With this view, and thanks to the flexible tip of the scope, it is possible, if desired, to ligate the splenic artery. The instruments are then moved to the posterior face of the spleen and the table is tilted to the left to take advantage of gravity and obtain exposure of the retrosplenic area. The posterior spleno-renal attachments are freed. Sometimes, especially if the umbilical approach is used and there are some difficulties with the more posterior and upper part of the upper splenic pole, a 3-mm instrument can be introduced through the left flank. This mininstrument can be used to retract or section (hook) retroperitoneal adhesions [Figure 2b]. Once the spleen is completely mobile, the flexible scope is retrieved and the intraabdominal visual control is changed to a 5-mm scope. If the multichannel has several large bore ports (Qadriport, Olympus), the 10-mm scope can be maintained. A probe inserted through the left 5-mm trocar raises the splenic hilum, providing sufficient space for the placement of the stapler. A stapler with a 6-cm white cartridge (Echelon, Ethicon Endo-Surgery) is inserted through a 12-mm trocar/port and advanced to the splenic fossa. After adjusting the jaws, the stapler is applied several times to sever the splenic hilum. Once the spleen is completely free, a 15-mm endobag (Endocatch II; Covidien, Mansfield, MA, USA) is inserted. The spleen is grasped with a 5-mm instrument and hung in the splenic fossa. The bag is deployed below the organ and the spleen is introduced. The bag is pulled to the umbilical incision and the spleen is retrieved intact or morcellated.

**Figure 2b:**
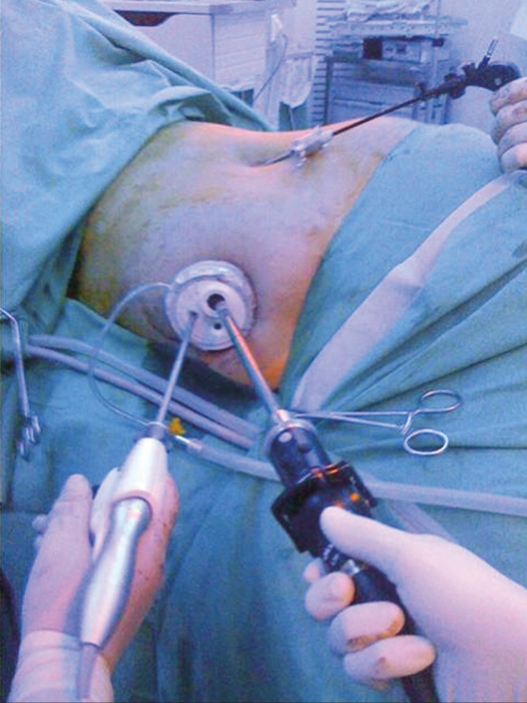
Intraoperative image of an hybrid single-port access splenectomy with the help of a 3-mm mininstrument

In the case of fenestration of a splenic cyst [Figures 3a and b], the first step is to puncture and evacuate the cyst content. Then, with the help of the harmonic scalpel, we excise the maximum segment of the wall cyst, reaching the spleen parenchyma. This very important step lessens the risk of cyst relapse if the remaining cyst wall is excessive and a residual cavity is left. Once hemostasis is completed, cyst wall fragments are extracted in an endobag.

**Figure 3a:**
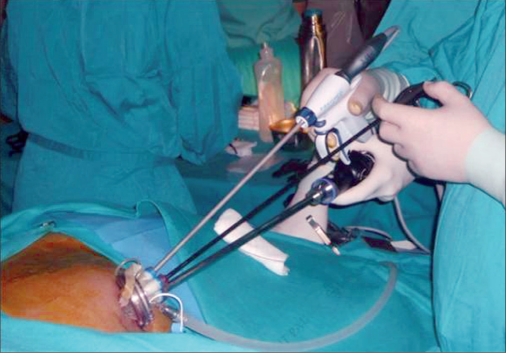
(a) Intraoperative image of a single-port access splenic cyst unroofing.

**Figure 3b:**
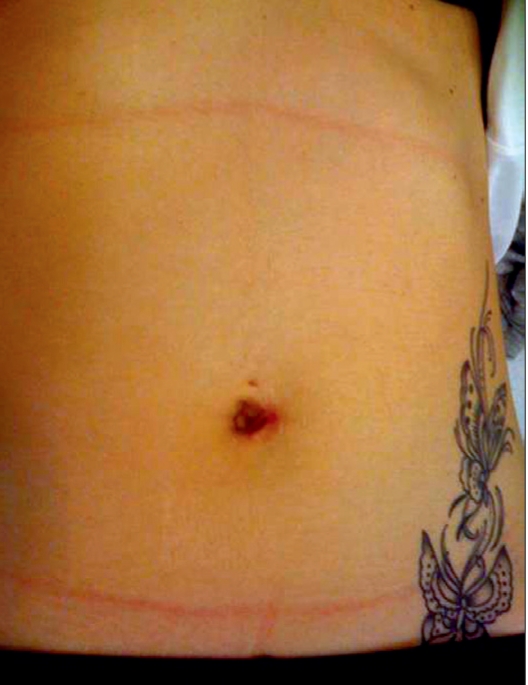
b: Postoperative aesthetic appeareance

## LITERATURE REVIEW

We reviewed all clinical reports published in PubMed and Google with the keywords: splenectomy, single-incision, single port, spleen and laparoscopy. We found eight papers, four of which were case reports.[7–13] There was only one series, and this included few cases. All the reports were published after January 2009. The summary of this cumulated series is plotted in Table 1.

**Table 1:** Publications to date

SPA splenectomy: World experience (PubMed, July 2010										

Author/year	n	Device	Position	Approach	BMI	Conversion	Blood loss	Op time	Stay	Spleeen weight
Barbaros/09	2	3 ports	Semilateral	Umbilical	??	no	??	??	48 h	??
Krukowsky/09	1	SILS	Lateral	Subcostal	??	no	??	??		??
Vatansev/09	1	2 ports	Semilateral	Umbilical	??	no	??	45’	36 h	??
Malladi/09	1	SILS	Semilateral	Umbilical	23	no	<10 cc	133’	48 h	¿?
Targarona/10	8	Several	Lateral	Umb/subc	24	2/8	<100 cc	97’	4 d	500
You/10	3	3 ports	Lateral	Subcostal	25	no	?	120’	??	??
Rottman/10	1	3 ports	Semilateral	Umbilicus	??	no	0	180	3	??
n	17									

## DISCUSSION

Preliminary results published to date indicate that the spleen can be safely removed using single-incision surgery and all the authors have unanimously endorsed the feasibility of this approach. However, available evidence is still scarce. It is based only on case reports and one small series with a total of 17 patient and, therefore, firm conclusions cannot yet be drawn. Besides, experience to date has raised a number of technical and clinical questions.

The surgical technique described is similar to the standard laparoscopic procedure, but some small variations have been suggested to solve the difficulties presented by SPA (clashing, lack of triangulation, odd angles and lack of space), with the surgeon handling the instruments and the assistant controlling the camera. Mallladi[6] suggests that the conflict between the camera and dissection instruments could be eased by the surgeon holding the camera while the assistant would manipulate the retraction instruments. In our experience, we have found that a flexible tip scope provides a better vision due to angulation possibilities and that the surgeon can use both operative hands.

There is clearly some controversy surrounding two key aspects: (1) the device used for SPA splenectomy and (2) the placement of the incision in the abdominal wall. In relation to the first topic, a number of different devices are currently available or are under investigation. We have used four types: a multi trocar (Curcillo), a Triport and a Quadriport (Olympus) and the Uno (Ethicon) system. All of them proved to be useful, but there is not yet sufficient experience to recommend one over another. In case of the spleen, however, devices that allow the introduction of 15-mm instruments (endobag) are recommended.

The placement of the incision is a crucial question. Ideally, SPA offers less aggression and better cosmesis This latter advantage nevertheless only applies if the incision is made in the umbilicus However, depending on the patient’s body mass index and the shape and size of the spleen, some dissection manoeuvres can be specially difficult or even impossible due to the oblique dissection line between the umbilicus and the upper part of the spleen. In this situation, there are two solutions: first, the device can be placed in a subcostal access incision, in which case the aesthetic advantage would be lost or, second, a hybrid approach can be adopted to help the dissection, using additional mini or fine calibre instrumentation. In our experience, we have attempted the umbilicus approach in very thin patients only and, in two cases, we had to convert to conventional LS to achieve visibility of the dissecting area.

There are a number of disposable and reusable flexing instruments specially designed for SPA. We have used the TEM instruments as good dissecting instruments as their slightly curved tip is suitable for intraabdominal coaxial movements. However, we await a new generation of specifically designed instrumentation that could facilitate the dissecting, grasping and retracting manoeuvres.

To date, experience with SPA splenectomy is scarce and the level of evidence is low. Preliminary results are encouraging, as we have said, but a number of questions remain to be answered, namely patient selection and surgical technique standardization. To address this important question, findings from experience so far indicate that SPA offers better cosmesis, greater pain control, fewer postoperative complications and enhanced patient recovery. However, clinical trials comparing standard LS, mininstruments LS[14] and SPA techniques are lacking and the technique has not been applied in a sufficient number of cases to determine possible morbidity associated with the longer incisions. More experience and comparative trials are needed to determine the exact role of this interesting new approach.
